# Supplementary material for: Histopathological and Virological Findings of a Penile Papilloma in a Japanese Stallion with Equus Caballus Papillomavirus 2 (EcPV2)
Source: Pathogens. 2024 Jul 19;13(7):597. doi: 10.3390/pathogens13070597 (PMC11279536; doi:10.3390/pathogens13070597)
Supplement: Supplementary file 1 [file pathogens-13-00597-s001.zip › Supplementary Figure S1.pdf]

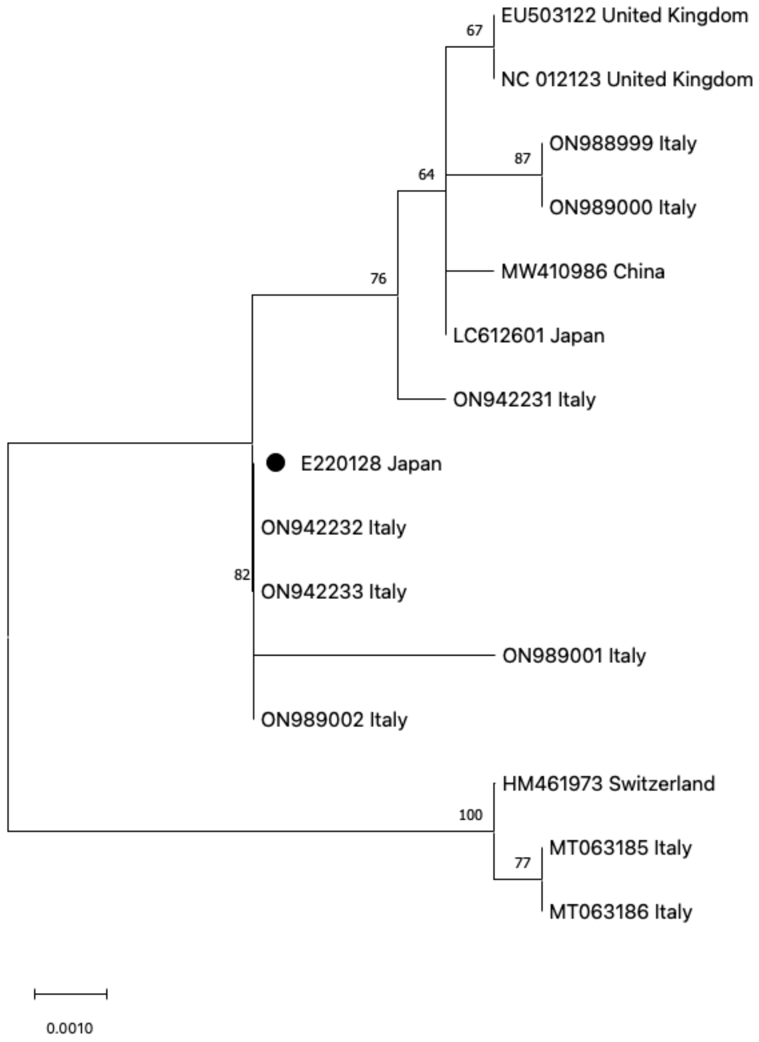

Supplementary Figure S1

A phylogenetic tree was constructed by the neighbor-joining method based on the L1 nucleotide sequence, 1500bp, with the current case (with black circle) and 14 EcPV2 strains. Origins of geographical area and GenBank accession numbers are noted. The bootstrap value of 1000 replicates is shown next to the branches.
