# Supplementary material for: Histopathological and Virological Findings of a Penile Papilloma in a Japanese Stallion with Equus Caballus Papillomavirus 2 (EcPV2)
Source: Pathogens. 2024 Jul 19;13(7):597. doi: 10.3390/pathogens13070597 (PMC11279536; doi:10.3390/pathogens13070597)
Supplement: Supplementary file 1 [file pathogens-13-00597-s001.zip › Supplementary Table S1.pdf]

Table S1 Used primers for detecting EcPV1, EvPV2, and BPV1, and sequening the EcPV2

Primer	Sequence (5'-3')	Location (nt)	Expected product size (bp)	Anneling temperature (°C)
**PV detection**				
498 EcPV1 L1 (forward)	GCATTTGCCCAGGCATTCAA	5737-5756	498	58
498 EcPV1 L1 (reverse)	TATCCACCGCCGTGACAAAA	6215-6234		
445 EcPV2 L1 (forward)	ATCAGTGTCAAGAAGGCGCA	6702-6721	445	54
445 EcPV2 L1 (reverse)	TTGGTGGGGCGCTTAGATTT	7156-7137		
BPV1/2 L1 subA modified (forward)	CCAGACTACCTCAAAATGGCTGA	BPV1 (L1, 6289-6311) , BPV2 (L1, 6282-6304)	436 (BPV1), 435(BPV2)	54
BPV1/2 L1 subA modified (reverse)	TAAAKGCTAGCTTATATTCTTCC	BPV1 (L1, 6702-6724), BPV2 (L1, 6695-6717)		
**EcPV2 whole genome sequencing**				
EcPV2 415 (forward)	CAGAAACGTGTACACTGCAAGAG	415-437	2701	55
EcPV2 3115 (reverse)	CAACGTACCCTCCCTTTTTGAAC	3115-3093		
EcPV2 2714 (forward)	TAAAAGGTTGTGGCAACGCTTAG	2714-2736	2992	55
EcPV2 5705 (reverse)	CAGGTGTTGTCTGTTCATTGTCC	5705-5683		
EcPV2 5264 (forward)	ACCATTGAGCTATCTGACCTGAC	5264-5286	3082	55
EcPV2 543 (reverse)	CCCTCGATCTCAATCCACCTTTT	543-521		
